# Larval swimming in the sea anemone *Nematostella vectensis* is sensitive to a broad light spectrum and exhibits a wavelength‐dependent behavioral switch

**DOI:** 10.1002/ece3.11222

**Published:** 2024-04-15

**Authors:** Emma Lilly, Meghan Muscala, Camilla R. Sharkey, Kyle J. McCulloch

**Affiliations:** ^1^ Department of Ecology, Evolution, and Behavior University of Minnesota St. Paul Minnesota USA

**Keywords:** Anthozoa, larval swimming, light behavior, *Nematostella vectensis*, opsin, sea anemone

## Abstract

In nearly all animals, light‐sensing mediated by opsin visual pigments is important for survival and reproduction. Eyeless light‐sensing systems, though vital for many animals, have received relatively less attention than forms with charismatic or complex eyes. Despite no single light‐sensing organ, the sea anemone *Nematostella vectensis* has 29 opsin genes and multiple light‐mediated behaviors throughout development and reproduction, suggesting a deceptively complex light‐sensing system. To characterize one aspect of this light‐sensing system, we analyzed larval swimming behavior at high wavelength resolution across the ultraviolet and visual spectrum. *N. vectensis* larvae respond to light at least from 315 to 650 nm, which is a broad sensitivity range even compared to many animals with complex eyes. Planktonic swimming is induced by ultraviolet (UV) and violet wavelengths until 420 nm. Between 420 and 430 nm a behavioral switch occurs where at wavelengths longer than 430 nm, larvae respond to light by swimming down. Swimming down toward the substrate is distinct from light avoidance, as animals do not exhibit positive or negative phototaxis at any wavelength tested. At wavelengths longer than 575 nm, animals in the water column take increasingly longer to respond and this behavior is more variable until 650 nm where larval response is no different from the dark, suggesting these longer wavelengths lie outside of their sensitivity range. Larval swimming is the only motile stage in the life history of *N. vectensis*, and increased planktonic swimming could lead to greater dispersal range in potentially damaging shallow environments with short‐wavelength light exposure. Longer wavelength environments may indicate more suitable substrates for metamorphosis into the polyp stage, where the individual will remain for the rest of its life. Future work will test whether this robust behavior is mediated by multiple opsins.

## Graphical abstract

Larval sea anemones sense light from UV to red without an eye. Wavelengths In the violet/UV range cause animals to enter the planktonic phase while wavelengths from blue to green to red cause larvae to swim down toward the substrate.

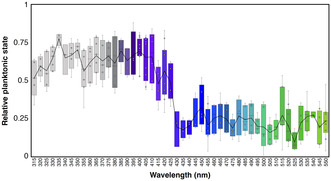

## 1 INTRODUCTION

Many animals use visual systems to detect light signals from the environment, conspecifics, or from other species (Land & Nilsson, 2012; Terakita & Nagata, 2014). The evolution and function of complex eyes have historically received the most attention, with much of our knowledge of visual function and evolution coming from a few focal eye types (Arendt et al., 2009; Fernald, 2006; Nilsson, 2013). However species and life stages with simple or no eyes are common, and animals with complex eyes have multiple non‐visual light‐sensing systems (Kaniewska et al., 2015; Nordström et al., 2003; Porter, 2016). Yet we know much less about the molecular, cellular, and behavioral aspects of these light‐sensing systems. Eyes likely evolved multiple times throughout evolution from more dispersed light‐sensing systems, and extant eyeless forms may hold important clues to understanding the repeated evolution of distinct eye types. To begin to address this major evolutionary question, we can ask how animals interface with the environment through behavior, as a first step in understanding light‐sensing traits in eyeless species.

Cnidaria (jellyfish, sea anemones, corals, etc.) are sister to Bilateria (vertebrates, arthropods, mollusks, etc.), making them an important phylogenetic comparison to better‐known visual systems. The lineage contains a wide range of visual system complexity, from eyeless to camera‐type eyes. Medusozoa, the group including jellyfish and *Hydra*, have evolved eyes at least 8 times from eyeless forms (Picciani et al., 2018). Mostly correlational evidence suggests cnidarian eyes share some homology with bilaterian visual systems in their development, photoreceptor proteins (opsins), and phototransduction pathways (Gehring, 2005; Hansen et al., 2023; Koyanagi et al., 2008; Kozmik et al., 2008, 2003). Some genetic links to light‐mediated behavior in non‐ocular light‐sensing have also been shown in Medusozoa. For instance, in the hydrozoan jellyfish *Clytia hemispherica*, a blue light‐absorbing opsin is responsible for oocyte maturation (Quiroga Artigas et al., 2018). *Hydra magnipapillata* are eyeless, yet evidence shows their contractile response and cnidocyte firing are regulated by light in a mechanism similar to some bilaterian phototransduction (Macias‐Munõz et al., 2019; Plachetzki et al., 2012, 2010; Vöcking et al., 2022).

Anthozoa, the other major cnidarian group including corals, sea pens, and sea anemones, are all eyeless. Almost nothing is known about any molecular, genetic, or cellular processes of light‐sensing in this group, and behavioral data on specific light stimuli are similarly limited. Despite this lack of data, light‐associated behaviors such as spawning are well‐known in this clade (Boch et al., 2011; Fritzenwanker & Technau, 2002; Kaniewska et al., 2015; Lin et al., 2021). Much anthozoan behavior work has focused on circadian rhythms in feeding activity and spawning (Hendricks et al., 2012; Reitzel et al., 2013; Tarrant et al., 2019). Previous work has mostly tested behaviors in adults under natural light or dark conditions (Bell et al., 2006; Fabricius & Klumpp, 1995; Sebens & DeRiemer, 1977), but these do not attempt to parse salient light signals, and few have tested multiple signals or light conditions (Leach & Reitzel, 2020; Mason et al., 2011). Recently it has been shown that many species in the group including anemones and corals, the Hexacorallia, have very high numbers of opsins (McCulloch et al., 2023). As opsins are the protein component of photoreceptors in animal vision and in many forms of non‐ocular light‐sensing, it is possible anemones and corals have unexplored complexity in their light‐sensing systems. Thus, specific visual cues should be tested to better characterize the potentially complex light‐mediated behavioral repertoire in Anthozoa at multiple life stages.

*Nematostella vectensis* is a model sea anemone (Anthozoa:Hexacorallia) whose external fertilization, rapid development, robust whole‐body regeneration, and high‐quality genomes make it amenable for laboratory study (Fletcher et al., 2023; Layden et al., 2016; Rentzsch et al., 2017; Zimmermann et al., 2020). *N. vectensis* is a shallow‐water, estuarine, burrowing anemone, and can reproduce sexually or asexually via transverse fission (Layden et al., 2016). In sexual reproduction at room temperature, fertilized eggs begin to divide after about 2 h, undergo gastrulation in under 24 h, and develop into swimming larvae by about 48 h. The swimming planula larva is the only dispersive stage of this otherwise sessile animal. After about a week the planula elongates, undergoes metamorphosis, and forms a polyp, a miniature adult form with 4 tentacles surrounding a single opening (mouth). The polyp burrows into soft, shallow substrate in brackish water marshes and estuaries after metamorphosis where it will remain for the rest of its life. The polyp grows and adds up to 16 total tentacles and becomes sexually mature in 3 months (Layden et al., 2016).

**FIGURE 1:**
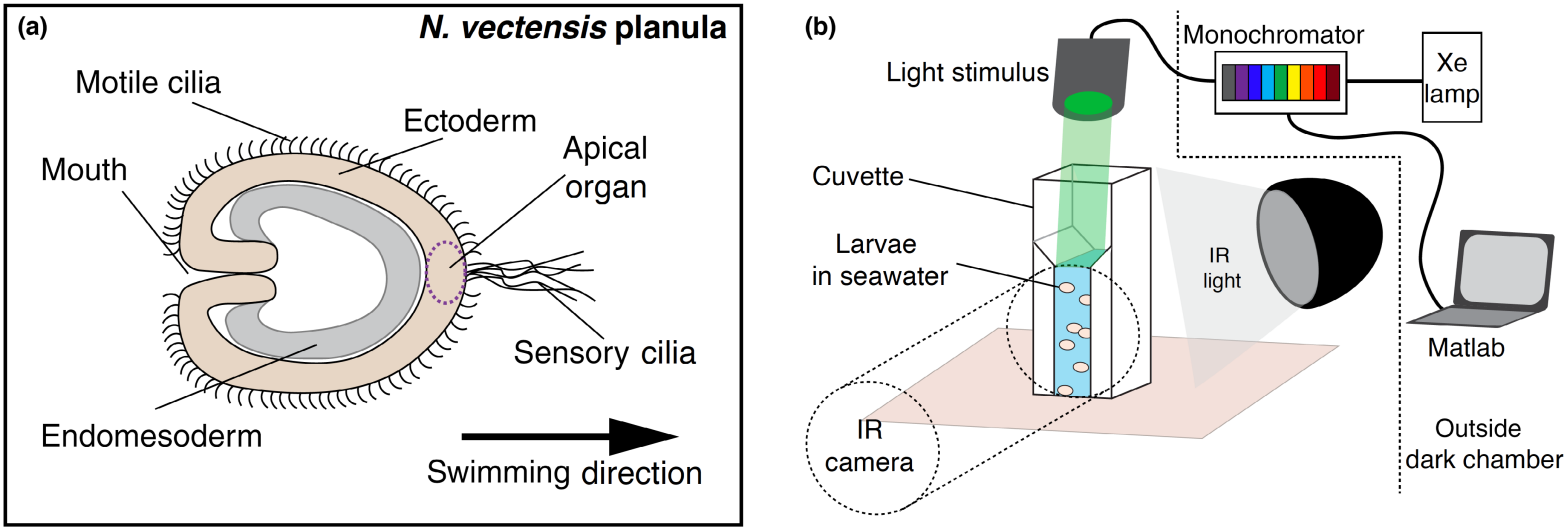
Larval anatomy and behavioral setup. (a) Planulae are diploblastic and covered uniformly in motile cilia. Swimming direction is opposite to the oral pole, with a sensory ciliated organ known as the apical organ at the front in the direction of swimming. (b) Experimental setup. Light from a xenon arc lamp passes through a monochromator controlled by a computer and is delivered via fiber optic cable into the dark chamber directly above the cuvette. The animals are bathed in infrared light and the infrared camera faces the cuvette from the side.

Therefore, as the planula swims in the water column, finding a suitable substrate is crucial for an individual's future survival and reproduction. To accomplish this task, the planula is uniformly covered in motile cilia that propel it through the water (Figure 1a), and it also has a sensory organ with much longer cilia called the apical organ located at the aboral (non‐mouth pole) ectoderm oriented forward in the direction of swimming (Hand & Uhlinger, 1992; Marinković et al., 2020). This apical organ expresses multiple homologous genes known in other species to be sensory‐related, including opsins (McCulloch et al., 2023; Rentzsch et al., 2008; Sinigaglia et al., 2015). Despite being eyeless, *N. vectensis* is typical among hexacorals in having among the most opsins of any animal, at 29 (McCulloch et al., 2023). The expression patterns of these opsins and the presence of genes involved in multiple bilaterian phototransduction pathways suggest a diversity of light‐mediated behavioral responses in *N. vectensis* (Hansen et al., 2023; McCulloch et al., 2023). For instance, some opsins are found only in adults and may be sexually dimorphic in expression levels, suggesting a role in reproduction, while others are only found in larval stages, suggesting a role in swimming and substrate finding in this species (McCulloch et al., 2023). However little is known about the sensory mechanisms required for substrate finding.

To begin to understand the connections between opsins and light‐sensing while swimming, we need a detailed understanding of the behavioral phenotype. Larval swimming in *N. vectensis* is known to increase with “disturbance” including to light, but this behavior is uncharacterized in the literature (Hand & Uhlinger, 1992). To characterize this light‐mediated behavior, we tested the response of *N. vectensis* larvae to 54 monochromatic wavelengths of light in *N. vectensis* larval swimming. We show *N. vectensis* larvae can detect wavelengths at least between 315 and 650 nm, and larvae respond to these colors (UV to red) in consistent and robust ways.

## 2 MATERIALS AND METHODS

### 2.1 Husbandry

*Nematostella vectensis* adults descended from the CH2xCH6 strain were kept in ⅓ concentration Instant Ocean seawater, at a constant temperature of 18°C, alternating light and dark exposure every 12 h. Adults were fed brine shrimp four to five times a week. To induce spawning, *N. vectensis* placed in containers were exposed to 24°C and white light for 6 h overnight. Eggs from multiple adults were collected in the morning, fertilized, and allowed to develop for 48 h at room temperature. At 2 days post‐fertilization larvae were placed in a petri dish in the dark at 18°C for at least 24 h before the experiment.

### 2.2 Short wavelength behavior experimental setup (315–550 nm)

Behavior experiments were conducted in a dark chamber with blackout curtains on an air table. See Figure 1 for the behavior setup schematic (Figure 1b). Hardware for the generation of light stimulus was located outside of the chamber and stimulus was delivered via fiber optic cable. The intensity and wavelength of light from a 150 W xenon arc lamp (Osram) were controlled using a monochromator (Optoscan, Cairn Research) and a DAQ board (USB‐6358, National Instruments), using custom MATLAB scripts (Data Acquisition Toolbox, Mathworks). Wavelength was determined by the angle of a 2400 line‐ruled diffraction grating (315–550 nm) or 1200 line‐ruled grating (450–700 nm) and intensity was adjusted by changing the width of monochromator in and out slits. The output light was measured at the approximate point of animal illumination using a spectrophotometer (Ocean FX, OceanOptics) calibrated for irradiance measurements (DH‐3P‐BAL‐CAL, OceanOptics). Photon flux and wavelength were measured and adjusted to yield isoquant stimuli (7.18 × 10^15^ photons cm^−2^ s^−1^), at each test wavelength. See Sharkey et al. (2020) for further information about the optical set up and monochromator calibration. Experiments were filmed from the side with a Minolta MN200 NV infrared video camera, with only infrared light for illumination at 30 frames per second.

**FIGURE 2:**
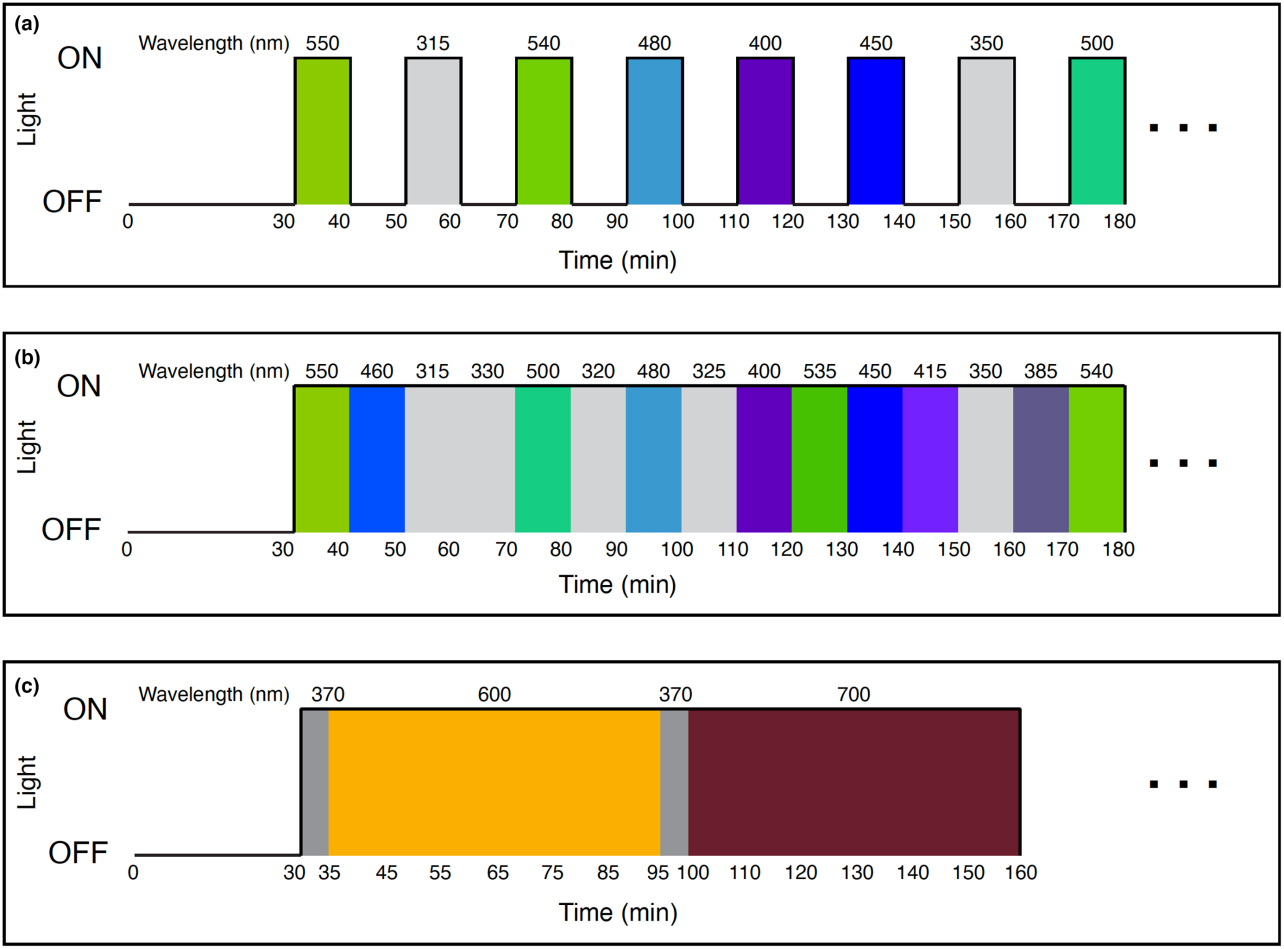
Light protocols used for short‐ and long‐wavelength experiments in this study. (a) Short‐wavelength light protocol with light and dark steps with semi‐randomized order of wavelengths. (b) Short‐wavelength light protocol with no dark steps. (c) Long‐wavelength protocol with hour long periods interspersed with UV light treatments to move animals back into the column. In all panels, time is in minutes, lights are colored and peak wavelengths are listed at the top corresponding to each light treatment. Ellipses signify experiments continued further than the examples shown with more colors.

Between 100 and 200 dark‐adapted larvae were added with seawater in a 10 × 4 × 45 mm acrylic cuvette (Sarstedt) with the light source directly above. Animals were allowed to settle in the cuvette in the dark for 30 min prior to the start of the experiment. The stage was lit for filming with an A14 infrared illuminator peaking at 850 nm (Tendelux). Dim red light was used to work in the dark room. Light was delivered automatically via Matlab using the Data Acquisition Toolbox (Mathworks), maintaining intensity (photon flux) while changing wavelength. Experiments either alternated between 10 min of dark and 10 min of light periods (Figure 2a), or continuous wavelength changes at 10 min periods with no dark periods (Figure 2b). Light behavior was indistinguishable between the two methods, so we combined averages from both for our short wavelength dataset. The long wavelength dataset alternated between UV and light or dark treatment in randomized order. For complete experimental information, all of our light protocols and raw tracked data are available on Dryad: https://doi.org/10.5061/dryad.wdbrv15vs. The order of wavelengths was semi‐randomized to ensure both short and long wavelengths for normalization (see below). The number of experiments per wavelength denotes the number of independent spawns that were tested at that wavelength. The same batch of animals was not re‐used for any single wavelength, but if we could not capture all wavelengths in 1 day due to time constraints we did use the same batch the next day to be able to get remaining untested wavelengths.

Pilot experiments were carried out before the dataset was collected, and temperature sensors were used to record changes in temperature over the course of the different wavelengths. The probe was placed in the seawater in the cuvette in identical conditions to our experimental dataset. No changes were noted in temperature at any wavelength measured to at least 0.01°C.

### 2.3 Long wavelength behavior experimental setup (575–700 nm)

Longer wavelength experiments were conducted using a similar setup as above with the following changes. The monochromator used to generate longer wavelengths was unable to also generate short‐wavelength light needed for the larvae to swim into the water column (see Section 3). To be sure that larvae were responding to longer wavelengths, we used an LED UV light controlled by a dual OptoLED power supply (Cairn Research) which peaked at 370 nm. The output of the LED was measured using a spectrophotometer at the point of the cuvette and manually adjusted until it was set to the same approximate total intensity as the long wavelengths presented to the animals. Animals were exposed to 5 min of UV light, then the test wavelength (575, 600, 625, 650, 675, or 700 nm), or darkness for 1 h (Figure 2). We saw no difference in swimming behavior in preliminary tests with 1 or 2 h in the dark, therefore only 1 h of darkness was used for experiments. For wavelengths 575 and 600 nm, behavioral response reached equilibrium (all animals swam to the bottom of the cuvette) well before an hour and some trials were stopped earlier than an hour.

### 2.4 Video analysis

**FIGURE 3:**
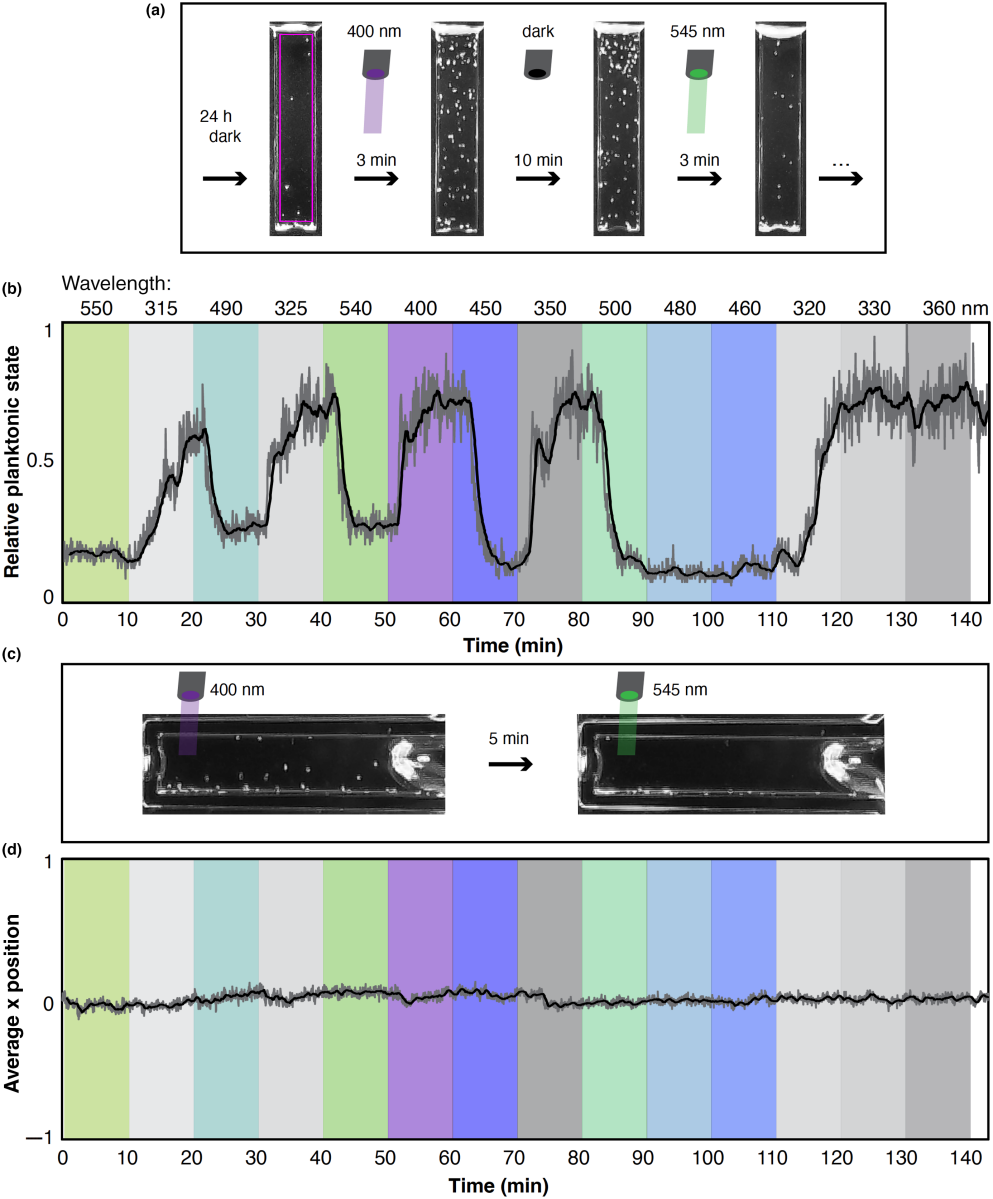
Experimental setup and example video analysis. (a) Example video frames analyzed by Trackmate, showing differences between light conditions. After being in the dark for 24 h, animals are not swimming in the water column. In UV light, swimming immediately increases within 3 min. Swimming activity is not changed in the dark following UV light after 10 min (or at least 2 h, see below). Green light causes nearly all larvae to swim down within 3 min. Pink box shows region of interest where swimming activity is tracked. (b) Example plotted after Trackmate analysis with wavelength treatments overlaid on the experiment in color. Downward swimming occurs with any wavelength over 430, higher relative planktonic swimming occurs at any wavelength shorter than 430. Dark gray line is raw data, black line is moving average over 20 previous datapoints. (c) Example of phototaxis setup under two light conditions. Animals do not swim on average toward or away from light source, but still show up and down swimming in the water column. (d) Example results from a single phototaxis experiment. Total of 7 experiments were conducted. Average × position of the spots calculated from Trackmate does not change and remains evenly distributed across × positions (near 0), regardless of wavelength.

**FIGURE 4:**
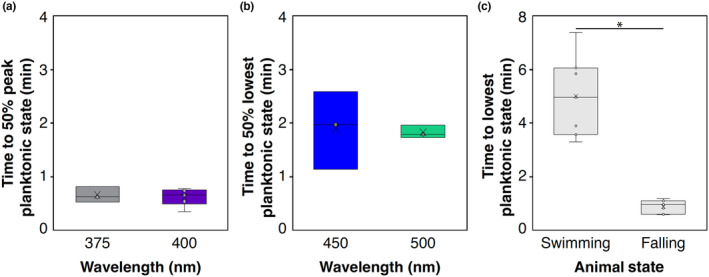
Timing of behavioral changes. Timing measured at 50% behavioral shifts from highest relative planktonic state to lowest relative planktonic state (substrate seeking) (a) or lowest to highest relative planktonic state (b) are shown, as estimated by sigmoid curve fits for representative wavelengths. (a) 375 nm, *N* = 4; 400 nm, *N* = 6. (b) 450 nm, *N* = 3; 500 nm, *N* = 3. (c) shows the time it takes animals to actively swim down (*N* = 7, wavelengths >430 nm ≤550 nm) versus the time it takes immobilized live animals to passively sink (*N* = 7). Times to reach substrate are significantly different (Welch's two sample *t*‐test, *p* < .001).

Video analysis of the swimming larvae was conducted using FIJI v2.9.0/1.53t and Trackmate v7.0 (Ershov et al., 2022; Schindelin et al., 2012). Examples of input video screenshots are shown in Figure 3a. Videos were imported by the ffmpeg plugin v4.2.2 in FIJI and decimated by 100 to reduce file size for analysis. The region of the cuvette to be analyzed was defined using the FIJI selection tool, avoiding the walls of the cuvette, the water surface, or the bottom where larvae are settled (see Figure 3a). Trackmate was run with default settings except for the quality threshold, which was changed to 2.4. Trackmate calculated the number of spots within the region of interest corresponding to each swimming larva anywhere in the water column, at every frame. The number of spots per frame was exported as a .csv file and then normalized by the peak spot number over the entire experiment to get relative planktonic level (i.e., 1 would indicate the most larvae swimming in the water column and 0 would indicate all larvae at the bottom of the cuvette). At all wavelengths, the longest it took to adjust from the previous condition and reach the new wavelength equilibrium was about 90 analysis frames (5 min, examples Figure 4). Therefore, averages were calculated after equilibrium was reached (after 90 frames). Averages from each replicate experiment and wavelength were then averaged and plotted as a box and whisker plot between 315 and 550 nm (see Appendix S1 for replicate numbers). After data collection during analysis, some data were removed if identical conditions were not met over the course of the entire video (e.g., cuvette was moved during trial, not positioned directly facing camera, too few or too many animals, etc.). To quantify significant differences in this large dataset, the data were tested for normality using a Shapiro–Wilk test (*W* = 0. 94,154, *p*‐value < .01), followed by a Kruskal‐Wallis rank sum test to determine if there was a significant effect of wavelength on the response (*χ* ^2^ = 217.82, df = 47, *p*‐value < .01). To determine which treatments were significantly different from one another a pairwise Wilcoxon rank sum exact test was performed with Benjamini–Hochberg (BH) correction for multiple tests (Table 1).

**TABLE 1:** Wilcoxon rank sum significance values for short wavelength swimming activities at equilibrium between 315 nm and 550 nm.

	315	320	325	330	335	340	345	350	355	360	365	370	375	380	385	390	395	400	405	410	415	420	425	430	435	440	445	450	455	460	465	470	475	480	485	490	495	500	505	510	515	520	525	530	535	540	545
320	0.4627	–	–	–	–	–	–	–	–	–	–	–	–	–	–	–	–	–	–	–	–	–	–	–	–	–	–	–	–	–	–	–	–	–	–	–	–	–	–	–	–	–	–	–	–	–	–
325	0.6944	0.813	–	–	–	–	–	–	–	–	–	–	–	–	–	–	–	–	–	–	–	–	–	–	–	–	–	–	–	–	–	–	–	–	–	–	–	–	–	–	–	–	–	–	–	–	–
330	0.13	0.4973	0.1762	–	–	–	–	–	–	–	–	–	–	–	–	–	–	–	–	–	–	–	–	–	–	–	–	–	–	–	–	–	–	–	–	–	–	–	–	–	–	–	–	–	–	–	–
335	0.0375	0.0375	0.027	0.0524	–	–	–	–	–	–	–	–	–	–	–	–	–	–	–	–	–	–	–	–	–	–	–	–	–	–	–	–	–	–	–	–	–	–	–	–	–	–	–	–	–	–	–
340	0.2366	0.7226	0.7027	1	0.1941	–	–	–	–	–	–	–	–	–	–	–	–	–	–	–	–	–	–	–	–	–	–	–	–	–	–	–	–	–	–	–	–	–	–	–	–	–	–	–	–	–	–
345	0.0625	0.3481	0.3797	0.9634	0.1139	1	–	–	–	–	–	–	–	–	–	–	–	–	–	–	–	–	–	–	–	–	–	–	–	–	–	–	–	–	–	–	–	–	–	–	–	–	–	–	–	–	–
350	0.0625	0.0625	0.0615	0.3196	0.1899	0.7226	0.7027	–	–	–	–	–	–	–	–	–	–	–	–	–	–	–	–	–	–	–	–	–	–	–	–	–	–	–	–	–	–	–	–	–	–	–	–	–	–	–	–
355	0.7919	1	0.9918	0.3958	0.0215	0.5412	0.3196	0.0615	–	–	–	–	–	–	–	–	–	–	–	–	–	–	–	–	–	–	–	–	–	–	–	–	–	–	–	–	–	–	–	–	–	–	–	–	–	–	–
360	0.4807	0.813	0.6461	0.8622	0.0426	0.9785	0.6944	0.3797	0.6	–	–	–	–	–	–	–	–	–	–	–	–	–	–	–	–	–	–	–	–	–	–	–	–	–	–	–	–	–	–	–	–	–	–	–	–	–	–
365	0.1841	0.5856	0.2291	0.8521	0.266	0.8165	1	0.9634	0.3291	0.6944	–	–	–	–	–	–	–	–	–	–	–	–	–	–	–	–	–	–	–	–	–	–	–	–	–	–	–	–	–	–	–	–	–	–	–	–	–
370	0.58	0.9396	0.4807	0.8842	0.3043	1	0.9396	0.8321	0.6939	0.9886	0.8842	–	–	–	–	–	–	–	–	–	–	–	–	–	–	–	–	–	–	–	–	–	–	–	–	–	–	–	–	–	–	–	–	–	–	–	–
375	0.0896	0.4069	0.2291	0.8521	0.0524	1	0.9634	0.5856	0.2155	0.7826	0.9785	0.8842	–	–	–	–	–	–	–	–	–	–	–	–	–	–	–	–	–	–	–	–	–	–	–	–	–	–	–	–	–	–	–	–	–	–	–
380	0.5853	1	0.9908	0.8622	0.393	0.8535	0.9035	0.6944	0.7826	1	0.6944	0.9886	0.9386	–	–	–	–	–	–	–	–	–	–	–	–	–	–	–	–	–	–	–	–	–	–	–	–	–	–	–	–	–	–	–	–	–	–
385	0.0625	0.1666	0.0969	0.4973	0.1899	0.5519	0.8321	1	0.13	0.4807	0.9634	0.8321	0.6939	0.6944	–	–	–	–	–	–	–	–	–	–	–	–	–	–	–	–	–	–	–	–	–	–	–	–	–	–	–	–	–	–	–	–	–
390	0.3043	0.7041	0.393	0.9032	0.0588	0.9488	0.8622	0.3043	0.3578	0.9803	0.8012	0.9785	0.6939	0.9803	0.7041	–	–	–	–	–	–	–	–	–	–	–	–	–	–	–	–	–	–	–	–	–	–	–	–	–	–	–	–	–	–	–	–
395	0.3043	0.7041	0.6372	0.9868	0.4973	0.9488	0.8622	0.7041	0.6939	0.8842	1	0.8622	0.9868	0.8842	0.7041	1	–	–	–	–	–	–	–	–	–	–	–	–	–	–	–	–	–	–	–	–	–	–	–	–	–	–	–	–	–	–	–
400	0.0184	0.0696	0.0781	0.4493	0.223	0.8652	0.5973	0.9094	0.0588	0.2904	0.8521	0.6697	0.5856	0.5422	0.9652	0.3987	0.5379	–	–	–	–	–	–	–	–	–	–	–	–	–	–	–	–	–	–	–	–	–	–	–	–	–	–	–	–	–	–
405	0.3043	0.6856	0.3501	0.761	0.2445	0.8622	0.9785	1	0.3316	0.8321	0.9386	0.9785	0.9386	0.8983	0.9785	0.6674	0.9917	0.9533	–	–	–	–	–	–	–	–	–	–	–	–	–	–	–	–	–	–	–	–	–	–	–	–	–	–	–	–	–
410	0.4807	0.6944	0.4627	0.8622	0.5057	0.9785	1	1	0.5232	0.742	1	0.9035	0.9918	0.8416	1	0.8842	0.8842	0.9269	0.9545	–	–	–	–	–	–	–	–	–	–	–	–	–	–	–	–	–	–	–	–	–	–	–	–	–	–	–	–
415	0.8321	0.1666	0.5853	0.0397	0.0375	0.2366	0.0625	0.0375	0.4069	0.212	0.0896	0.2469	0.0397	0.4807	0.0625	0.1899	0.1139	0.0184	0.1464	0.212	–	–	–	–	–	–	–	–	–	–	–	–	–	–	–	–	–	–	–	–	–	–	–	–	–	–	–
420	0.736	0.8739	1	0.4807	0.0746	0.813	0.4743	0.2397	1	0.6209	0.3318	0.5964	0.4807	0.813	0.2397	0.5705	0.6502	0.1401	0.3578	0.3926	0.5964	–	–	–	–	–	–	–	–	–	–	–	–	–	–	–	–	–	–	–	–	–	–	–	–	–	–
425	0.6939	0.2446	0.5232	0.0719	0.0215	0.1979	0.0397	0.0283	0.472	0.13	0.0566	0.2446	0.0307	0.2915	0.0397	0.1882	0.0796	0.0103	0.0786	0.13	1	0.5853	–	–	–	–	–	–	–	–	–	–	–	–	–	–	–	–	–	–	–	–	–	–	–	–	–
430	0.0682	0.0682	0.052	0.0382	0.103	0.1743	0.0682	0.0682	0.0382	0.052	0.0382	0.0682	0.0382	0.052	0.0682	0.103	0.103	0.0185	0.027	0.052	0.0682	0.0305	0.0382	–	–	–	–	–	–	–	–	–	–	–	–	–	–	–	–	–	–	–	–	–	–	–	–
435	0.0375	0.0375	0.027	0.0215	0.0588	0.103	0.0375	0.0375	0.0215	0.027	0.0215	0.0375	0.0215	0.027	0.0375	0.0588	0.0588	0.0103	0.0144	0.027	0.0375	0.0118	0.0215	0.9488	–	–	–	–	–	–	–	–	–	–	–	–	–	–	–	–	–	–	–	–	–	–	–
440	0.0375	0.0375	0.027	0.0215	0.0588	0.103	0.0375	0.0375	0.0215	0.027	0.0215	0.0375	0.0215	0.027	0.0375	0.0588	0.0588	0.0103	0.0144	0.027	0.0375	0.0118	0.0215	1	0.8317	–	–	–	–	–	–	–	–	–	–	–	–	–	–	–	–	–	–	–	–	–	–
445	0.0625	0.0375	0.027	0.0215	0.0588	0.103	0.0375	0.0375	0.0328	0.027	0.0215	0.0375	0.0215	0.0426	0.0375	0.0588	0.0588	0.0103	0.0144	0.027	0.0625	0.0286	0.0524	0.9488	0.1941	0.8317	–	–	–	–	–	–	–	–	–	–	–	–	–	–	–	–	–	–	–	–	–
450	0.1666	0.0375	0.0393	0.0138	0.0375	0.0682	0.0258	0.0258	0.0615	0.0393	0.0206	0.0375	0.0138	0.0615	0.0258	0.0375	0.0625	0.0089	0.0184	0.0393	0.1666	0.0588	0.13	0.5519	0.1899	0.1899	0.7041	–	–	–	–	–	–	–	–	–	–	–	–	–	–	–	–	–	–	–	–
455	0.0218	0.0118	0.0095	0.0089	0.0184	0.0328	0.0118	0.0118	0.0105	0.0095	0.0089	0.0118	0.0089	0.0095	0.0118	0.0184	0.0184	0.0045	0.0064	0.0095	0.0218	0.007	0.0138	0.9868	0.9161	0.8317	0.2506	0.2774	–	–	–	–	–	–	–	–	–	–	–	–	–	–	–	–	–	–	–
460	0.027	0.0184	0.0138	0.0103	0.027	0.052	0.0184	0.0184	0.0261	0.0138	0.0103	0.0184	0.0103	0.0138	0.0184	0.027	0.027	0.0064	0.0095	0.0138	0.0615	0.0103	0.0261	0.7027	0.5057	0.6372	0.9803	0.5853	0.813	–	–	–	–	–	–	–	–	–	–	–	–	–	–	–	–	–	–
465	0.0283	0.0138	0.0103	0.0095	0.0215	0.0382	0.0138	0.0138	0.0184	0.0103	0.0095	0.0138	0.0095	0.0138	0.0138	0.0215	0.0215	0.0058	0.0068	0.0103	0.0397	0.0103	0.0243	0.5412	0.3578	0.5705	0.9868	0.6939	0.426	0.8622	–	–	–	–	–	–	–	–	–	–	–	–	–	–	–	–	–
470	0.0625	0.0375	0.027	0.0215	0.0588	0.103	0.0375	0.0375	0.0328	0.027	0.0215	0.0375	0.0215	0.027	0.0375	0.0588	0.0588	0.0103	0.0144	0.027	0.0625	0.0215	0.0328	0.7826	0.6461	0.8317	0.8317	0.7041	0.9904	0.9803	0.9032	–	–	–	–	–	–	–	–	–	–	–	–	–	–	–	–
475	0.0258	0.0258	0.0184	0.0138	0.0375	0.0682	0.0258	0.0258	0.0138	0.0184	0.0138	0.0258	0.0138	0.0184	0.0258	0.0375	0.0375	0.0089	0.0103	0.0184	0.0258	0.0095	0.0138	0.9032	0.9785	0.7041	0.3043	0.1666	0.9386	0.4807	0.3196	0.7041	–	–	–	–	–	–	–	–	–	–	–	–	–	–	–
480	0.0625	0.0375	0.027	0.0215	0.0588	0.103	0.0375	0.0375	0.0328	0.027	0.0215	0.0375	0.0215	0.027	0.0375	0.0588	0.0588	0.0103	0.0144	0.027	0.0625	0.0286	0.0524	0.558	0.4973	0.4973	1	0.8622	0.6193	1	0.9868	1	0.5705	–	–	–	–	–	–	–	–	–	–	–	–	–	–
485	0.027	0.0184	0.0138	0.0103	0.027	0.052	0.0184	0.0184	0.0194	0.0138	0.0103	0.0184	0.0103	0.0138	0.0184	0.027	0.027	0.0064	0.0095	0.0138	0.0393	0.0095	0.0194	0.8535	0.5057	0.7631	0.7631	0.4807	0.4983	0.9257	0.7826	1	0.3797	0.8842	–	–	–	–	–	–	–	–	–	–	–	–	–
490	0.0393	0.0184	0.0138	0.0103	0.027	0.052	0.0184	0.0184	0.0261	0.0138	0.0103	0.0184	0.0103	0.0138	0.0184	0.027	0.027	0.0064	0.0095	0.0138	0.0615	0.0103	0.0261	0.7027	0.5057	0.8842	0.8842	0.5853	0.813	0.9908	0.9386	0.9803	0.4807	1	0.9908	–	–	–	–	–	–	–	–	–	–	–	–
495	0.0184	0.0184	0.0138	0.0103	0.027	0.052	0.0184	0.0184	0.0103	0.0138	0.0103	0.0184	0.0103	0.0138	0.0184	0.027	0.027	0.0064	0.0095	0.0138	0.0184	0.0068	0.0103	0.9785	0.9803	0.9803	0.2778	0.212	0.9488	0.6461	0.363	0.6372	0.9035	0.5057	0.6461	0.5527	–	–	–	–	–	–	–	–	–	–	–
500	0.0375	0.0258	0.0184	0.0138	0.0375	0.0682	0.0258	0.0258	0.0283	0.0184	0.0138	0.0258	0.0138	0.0184	0.0258	0.0375	0.0375	0.0089	0.0103	0.0184	0.0625	0.0138	0.0206	1	1	1	0.5705	0.3481	0.7773	0.5853	0.5856	0.7041	1	0.8622	0.6944	0.5853	0.9035	–	–	–	–	–	–	–	–	–	–
505	0.0138	0.0138	0.0103	0.0095	0.0215	0.0382	0.0138	0.0138	0.0095	0.0103	0.0095	0.0138	0.0095	0.0103	0.0138	0.0215	0.0215	0.0058	0.0068	0.0103	0.0138	0.0064	0.0095	0.8165	0.9868	0.3578	0.1298	0.0615	0.9011	0.1762	0.0985	0.469	0.7919	0.1882	0.2291	0.2291	0.6	0.8842	–	–	–	–	–	–	–	–	–
510	0.0258	0.0258	0.0184	0.0138	0.0375	0.0682	0.0258	0.0258	0.0138	0.0184	0.0138	0.0258	0.0138	0.0184	0.0258	0.0375	0.0375	0.0089	0.0103	0.0184	0.0258	0.0095	0.0138	0.9032	0.9785	0.7041	0.4309	0.2469	0.9386	0.2872	0.3196	0.5705	1	0.4309	0.3797	0.3797	0.9035	0.9396	0.6939	–	–	–	–	–	–	–	–
515	0.0615	0.0206	0.0261	0.0095	0.0215	0.0382	0.0206	0.0138	0.0243	0.0194	0.0103	0.0283	0.0103	0.0375	0.0138	0.0328	0.0328	0.0068	0.0138	0.0261	0.0896	0.0215	0.0719	1	0.469	1	0.9868	0.6939	0.2485	1	0.9785	0.9032	0.5856	0.9032	1	0.8622	0.5232	0.5856	0.2155	0.3196	–	–	–	–	–	–	–
520	0.0184	0.0103	0.0103	0.0068	0.0144	0.027	0.0103	0.0103	0.0099	0.0095	0.0068	0.0103	0.0068	0.0103	0.0103	0.0144	0.0144	0.0038	0.0058	0.0095	0.0243	0.0068	0.0105	1	0.9917	0.7589	0.4817	0.3043	0.8739	0.547	0.3872	0.5716	0.9083	0.7589	0.4807	0.4807	0.8321	1	0.8842	1	0.4493	–	–	–	–	–	–
525	0.0375	0.0375	0.027	0.0215	0.0588	0.103	0.0375	0.0375	0.0215	0.027	0.0215	0.0375	0.0215	0.027	0.0375	0.0588	0.0588	0.0103	0.0144	0.027	0.0375	0.0118	0.0215	0.558	0.1941	0.103	0.0588	0.0625	0.3364	0.072	0.0524	0.1941	0.1899	0.103	0.072	0.072	0.1194	0.5705	0.266	0.1139	0.1298	0.1842	–	–	–	–	–
530	0.0625	0.0375	0.027	0.0215	0.0588	0.103	0.0375	0.0375	0.0328	0.027	0.0215	0.0375	0.0215	0.027	0.0375	0.0588	0.0588	0.0103	0.0144	0.027	0.0625	0.015	0.0328	0.9488	0.8317	0.9709	0.6461	0.5705	0.8317	0.9803	0.8012	0.9709	0.8622	0.8317	1	0.8842	0.9803	0.9785	0.469	0.9785	0.9032	0.9317	0.3191	–	–	–	–
535	0.0206	0.0138	0.0103	0.0095	0.0215	0.0382	0.0138	0.0138	0.0103	0.0103	0.0095	0.0138	0.0095	0.0138	0.0138	0.0215	0.0215	0.0058	0.0068	0.0103	0.0206	0.0103	0.0138	0.5412	0.266	0.3578	0.9868	0.7919	0.5555	0.9918	1	1	0.3196	1	0.9386	1	0.4441	0.6939	0.0985	0.2446	0.9785	0.3872	0.0524	0.8012	–	–	–
540	0.0682	0.0682	0.052	0.0382	0.103	0.1743	0.0682	0.0682	0.0382	0.052	0.0382	0.0682	0.0382	0.052	0.0682	0.103	0.103	0.0185	0.027	0.052	0.0682	0.0305	0.0382	0.8418	0.103	0.103	0.9488	0.9032	0.4232	1	1	1	0.2366	0.9488	0.7027	1	0.1666	0.9032	0.0382	0.2366	0.5412	0.5302	0.103	0.9488	1	–	–
545	0.0159	0.0118	0.0095	0.0089	0.0184	0.0328	0.0118	0.0118	0.0095	0.0095	0.0089	0.0118	0.0089	0.0095	0.0118	0.0184	0.0184	0.0045	0.0064	0.0095	0.0218	0.0064	0.0095	1	0.9161	0.9161	0.525	0.2149	1	0.4983	0.3597	0.8317	1	0.4277	0.6514	0.4276	0.9956	1	0.6944	0.9918	0.5555	0.9228	0.1882	0.9161	0.4892	0.5338	–
550	0.0144	0.0095	0.0103	0.0064	0.0138	0.0243	0.0095	0.0095	0.0095	0.0095	0.0075	0.0111	0.0064	0.0103	0.0095	0.0138	0.0138	0.0035	0.0064	0.0095	0.0144	0.0068	0.0105	0.9908	0.4555	0.6962	0.9918	0.5283	0.6189	0.7902	0.7565	0.9396	0.3262	0.6962	0.9634	0.8535	0.5853	0.8902	0.223	0.4627	1	0.558	0.1001	0.9918	0.6944	0.5283	0.7233

*Note*: Blue‐shaded cells are significantly different comparisons (*p* < .5).

To show that the response of larvae to darkness was not the same as lack of short wavelengths, we binned examples from our entire dataset where wavelengths went from short to long (*N* = 9), or long to short (*N* = 7), with dark periods in between. “Short” was defined as any wavelength shorter than 420 nm, and “long” was defined as any wavelength longer than 430 nm. The average planktonic level from the dark periods immediately preceding and following were compared with the activity for either the long or short wavelength in between. A Shapiro–Wilk test showed both short‐to‐long (*W* = 0.81547, *p*‐value < .01) and long‐to‐short (*W* = 0.81731, *p*‐value = .01) datasets were non‐normally distributed, and Kruskal–Wallis tests showed both had significant differences in the data (short‐to‐long, *χ* ^2^ = 17.429, df = 2, *p*‐value < .01; long‐to‐short, *χ* ^2^ = 13.781, df = 2, *p*‐value = .01). Wilcoxon rank sum exact tests were performed with Benjamini–Hochberg correction for multiple tests for each condition (Tables 2 and 3).

**TABLE 2:** Wilcoxon rank sum significance values comparing dark swimming activity before and after short wavelength light stimulus.

	Dark 1	Light 2 (SW)	Dark 2
Light 2 (SW)	0.00087	–	–
Dark 2	0.00087	0.45944	–
Light 1 (LW)	0.80478	0.00087	0.00087

*Note*: Blue‐shaded cells are significantly different comparisons (*p* < .5).

**TABLE 3:** Wilcoxon rank sum significance values comparing dark swimming activity before and after longer wavelength light stimulus.

	Dark 2	Dark 1	Light 2 (LW)
Dark 1	6.20E‐05	–	–
Light 2 (LW)	0.65	6.20E‐05	–
Light 1 (SW)	6.20E‐05	0.93	6.20E‐05

*Note*: Blue‐shaded cells are significantly different comparisons (*p* < .5).

To calculate half time to peak or lowest planktonic swimming state, planktonic swimming level over time for each trial was fitted with a sigmoid curve using non‐linear least squares (nls) regression, using the function:

$$F\left(x\right)=A+\frac{B-A}{1+e^{\left(\frac{x-C}{D}\right)}}$$

where the minimum asymptote (*A*), the maximum asymptote (*B*), the *x*‐value at the midpoint between *A* and *B* (*C*), and the slope value (*D*) were all predicted given the data. We used the value for *C* to estimate halfway to settling time and plotted these values as box plots. At wavelengths greater than 650 nm, behavior was variable and often unresponsive, so sigmoid fits were not appropriate. We compared half‐times to exit the planktonic state at longer wavelengths (575, 600, 625, and 650 nm) with representative shorter wavelength downward swimming responses estimated by the same method (450 and 500 nm). The Shapiro–Wilk test for normality (*W* = 0.77472, *p*‐value < .01) showed the data were not normally distributed. A Kruskal–Wallis rank sum test was performed (*χ* ^2^ = 21.557, df = 5, *p*‐value < .01), followed by a pairwise Wilcoxon rank sum exact test with BH correction to determine which treatments were significantly different from one another (Table 4). To calculate timing differences between swimming down and passive falling, the frame where the value for the sigmoid function first diverged from the minimum asymptote (*A*) was used. These data (*N* = 7) were tested for normality using a Shapiro–Wilk test (swimming: *W* = 0.93427, *p*‐value > .05; falling: *W* = 0.9162, *p*‐value > .05), and had unequal variances (Levene's test, df = 1, *F*‐value = 13.108, *p*‐value < .05), and a Welch's two‐sample *t*‐test was performed to determine whether the two groups were significantly different (*t*‐value = 7.1292, df = 6.2839, *p*‐value < .001).

**TABLE 4:** Wilcoxon rank sum significance values for comparisons of half times to down state at different wavelengths.

	450	500	575	600	625
500	0.808	–	–	–	–
575	0.969	1	–	–	–
600	0.049	0.049	0.043	–	–
625	0.049	0.049	0.032	0.049	–
650	0.049	0.049	0.032	0.049	0.278

*Note*: Blue‐shaded cells are significantly different comparisons (*p* < .05).

To quantify lack of responses at longer wavelengths, for each trial, the number of spots per frame for the last 20 frames was averaged and subtracted from the average number of spots from the first 20 frames at each wavelength. This yielded a change in swimming activity after 1 h of light exposure. These data were plotted as box plots and compared similarly to above. The Shapiro–Wilk test for normality (*W* = 0.89426, *p*‐value < .01) showed the data were not normally distributed. A Kruskal–Wallis rank sum test was used to determine if there were significant differences among treatments in the data, which there were (*χ* ^2^ = 14.955, df = 6, *p*‐value < .05). To determine which treatments were significantly different from one another a pairwise Wilcoxon rank sum test was performed with BH correction for multiple tests (Table 5).

**TABLE 5:** Wilcoxon rank sum significance values comparing initial and final swimming activities at long wavelengths.

	575	600	625	650	675	700
600	0.979	–	–	–	–	–
625	0.869	1	–	–	–	–
650	0.212	0.227	0.359	–	–	–
675	0.227	0.246	0.6	1	–	–
700	0.331	0.359	0.359	1	1	–
Dark	0.049	0.053	0.227	0.359	0.549	0.246

*Note*: Blue‐shaded cells are significantly different comparisons (*p* < .05).

### 2.5 Phototaxis experiments

Phototaxis experiments were conducted as above, but with the cuvette turned on its side. The light stimulus was applied at one end of the cuvette (Figure 3c). Larvae were allowed to spread across the full length of the cuvette in the dark for 30 min. After 30 min, the light protocol was started, and larval behavior was recorded in infrared. Video analysis was similar to above, but instead of total number of spots, the average × position of total spots for any given wavelength was used from the Trackmate output data.

## 3 RESULTS

We conducted a multi‐spectral behavioral experiment to test the effect of light on the swimming behavior of larval *N. vectensis*. Screenshots of typical videos and the region analyzed (pink box) are shown in Figure 3a. Throughout all experiments, animals switched between entering the water column or exiting by swimming down toward the substrate, which we define here as high and low levels of planktonic swimming. When presented with light between 315 and 420 nm, the number of animals entering the water column quickly increased and reached an equilibrium for the rest of the light presentation (Figure 3), which we define as the peak planktonic state. This movement was not directionally toward the light or surface, but animals consistently moved in all directions throughout the water column. When animals were presented with light from ~430 to 625 nm, they actively swam down to the bottom of the cuvette and continued swimming at the bottom, exiting the planktonic state and seeking substrate. Swimming behavior is identifiable by the spiral motion of animals and they often switch directions, as opposed to passive sinking (Videos 1 and 2). In contrast, after 24 h of darkness, animals were not seen in the water column and were less active in general. Under longer wavelengths when animals exited the planktonic phase, we could see animals were still actively swimming at the bottom of the cuvette but could not quantify this due to limitations in visualizing this in our setup.

**VIDEO 1:** Video showing larvae transition from lowest planktonic state in the dark, to peak planktonic swimming level under UV light (370 nm) then swimming to lowest planktonic swimming level under green light (550 nm).

**VIDEO 2:** Video showing larvae immobilized with MgCl_2_ passively sinking in the cuvette.

To further characterize and formally quantify this behavior, we measured photo‐activity at 5 nm intervals under illumination from 315 to 550 nm. We first noticed visually obvious changes in planktonic swimming levels between short (<425 nm) and longer (>425 nm) wavelengths (Figure 3a,b). The behavioral response to either short or long wavelength was robust regardless of light/dark combinations, order of wavelengths, and up to at least 5 h of testing (Figure 3b). Animals in the planktonic state were found within the region of interest in the water column (pink box, Figure 3a) and were tracked, contributing to higher planktonic swimming levels. When animals exited the planktonic state at the bottom of the cuvette, they exited the region of interest and therefore were not tracked, lowering the planktonic swimming level (Figure 3b).

We additionally wanted to know whether animals were moving toward or away from light (phototaxis). To assess whether swimming behavior was phototactic, the cuvette was placed on its side with the light on at one end (Figure 3c). Over a total of seven experiments, animals did not move toward or away from the light in any wavelength tested, suggesting no phototaxis. Photo‐activity (i.e., active swimming in the water column) was still present, similar to when the cuvette was upright only (Figure 3c,d).

**FIGURE 5:**
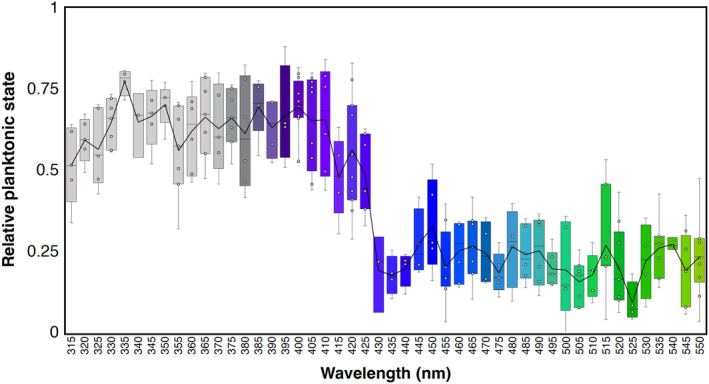
Relative planktonic swimming activity and switch from 315 to 550 nm. Boxplot of median planktonic swimming levels by wavelength. Line connecting each wavelength represents the mean. Relative planktonic swimming level at each wavelength was averaged over a minimum three experiments and up to 12 experiments. A large decrease in planktonic swimming is seen between wavelengths 420 and 430. Number of experiments for each wavelength are shown in Appendix S1.

To calculate relative levels of planktonic swimming activity under each light condition, we allowed larvae to achieve equilibrium, that is, when the change in number of tracked larvae over time plateaued. This allowed us to precisely estimate the half‐time to reach either the peak or lowest planktonic state (depending on wavelength). Larvae take approximately 1 min to reach halfway to peak planktonic state under shorter wavelengths, while the time to 50% of the lowest planktonic state is 2 min under longer wavelengths (Figure 4a,b). Animals responded by swimming up in wavelengths as short as 315 nm (limit of monochromator) (Figure 5). Between 335 and 415 nm, animals were at a similar peak planktonic state, and not significantly different from one wavelength to another (Table 1). Interestingly, peak planktonic state at 335 nm is significantly higher than most other wavelengths, and wavelengths 315–325 nm are lower than some other short‐wavelength planktonic levels (Figure 5, Table 1). At about 415 nm, planktonic swimming begins to decrease until about 425 nm, when a sudden decrease in planktonic swimming occurs. At 425 nm there is a switch in behavior where larvae respond by actively swimming down toward the substrate (Figure 5). Between 415 and 425 nm, patchy significant differences with both short and long wavelengths suggest a transition zone between up and down swimming under blue‐violet light (Figure 5, Table 1). Between 430 and 550 nm lowest planktonic states are not significantly different from one wavelength to another (Figure 5, Table 1). Lowest planktonic activity is not zero because at any given time, individuals may “jump” up into the water column and are counted, but then quickly descend back to the bottom.

**FIGURE 6:**
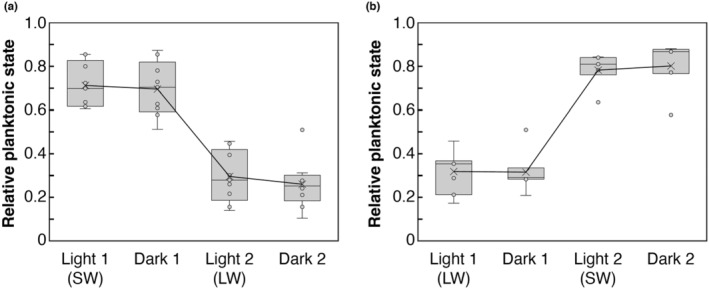
Short‐term dark behavior is dictated by previous light stimulus. (a) Average planktonic swimming level is shown at the end of each 10‐min period that followed the sequence: short wavelength light 1, dark period 1, long wavelength light 2, and dark period 2. The first light stimulus and dark period are significantly different from the second light period and second dark period (*N* = 9, Wilcoxon rank sum test, *p*‐value < .001). (b) Plot shows the reverse light sequence from (a). The first light stimulus and dark period are significantly different from the second light period and second dark period (*N* = 7, Wilcoxon rank sum test, *p*‐value < .001). All trials with the sequence: LW 1, dark, SW, dark were binned and plotted. SW, short wavelength between 315 and 415 nm; LW, long wavelength between 430 and 550 nm; line shows means for each light treatment. See Section 2 and Tables 2 and 3 for statistical tests and *p*‐value tables.

It is possible animals were responding to only shorter wavelengths and moving downward could be due to an absence of the salient signal, that is, long wavelengths are similar to darkness and animals swim down to the bottom and stay there. First, we show swimming down is distinct from passive falling qualitatively (Videos 1 and 2) and quantitatively. Figure 4c shows that immobilized live animals passively fall to the bottom of the cuvette in under 1 min with little variance, while swimming animals do not swim straight down and take much longer, at about 5 min with a much larger variance (Levene's test, df = 1, *F*‐value = 13.108, *p*‐value < .05; Welch's *t*‐test, *t*‐value = 7.1292, df = 6.2839, *p*‐value < .001). To show that animals were in fact actively responding by swimming to both short and long wavelengths tested, we compared dark levels of swimming activity before and after light treatments. To show this, we binned all examples from our dataset where the sequence of stimuli tested went from short wavelength (315–415 nm) to dark, to long wavelength (430–550 nm), to dark again, or vice versa (long to dark to short to dark). The initial light stimulus would prime the animals to be at either the peak or lowest planktonic state, and then the next stimulus should cause the opposite behavior, providing for the greatest change in swimming activity between any two stimuli. If downward swimming behavior in long wavelengths was similar to that in darkness, then we should see no difference in dark periods before or after long‐wavelength stimuli. Similarly, if the absence of light was causing animals to swim downwards then there should be a significant difference between dark periods before and after short wavelength stimuli. We show in both down‐to‐up and up‐to‐down sequences, planktonic swimming level in the dark does not significantly change after any light stimulus (Wilcoxon rank sum test, *p*‐value = 1; Tables 2 and 3), while planktonic swimming level in the dark preceding the light stimulus is significantly different for short (Wilcoxon rank sum test, *p* < .001; Figure 6, Table 2) and long wavelengths (Wilcoxon rank sum test, *p* < .01; Figure 6, Tables 2 and 3). This shows that swimming behavior in the dark is dictated by the previous wavelength tested, and therefore animals sense and alter their behavior in response to both short and long wavelengths.

**FIGURE 7:**
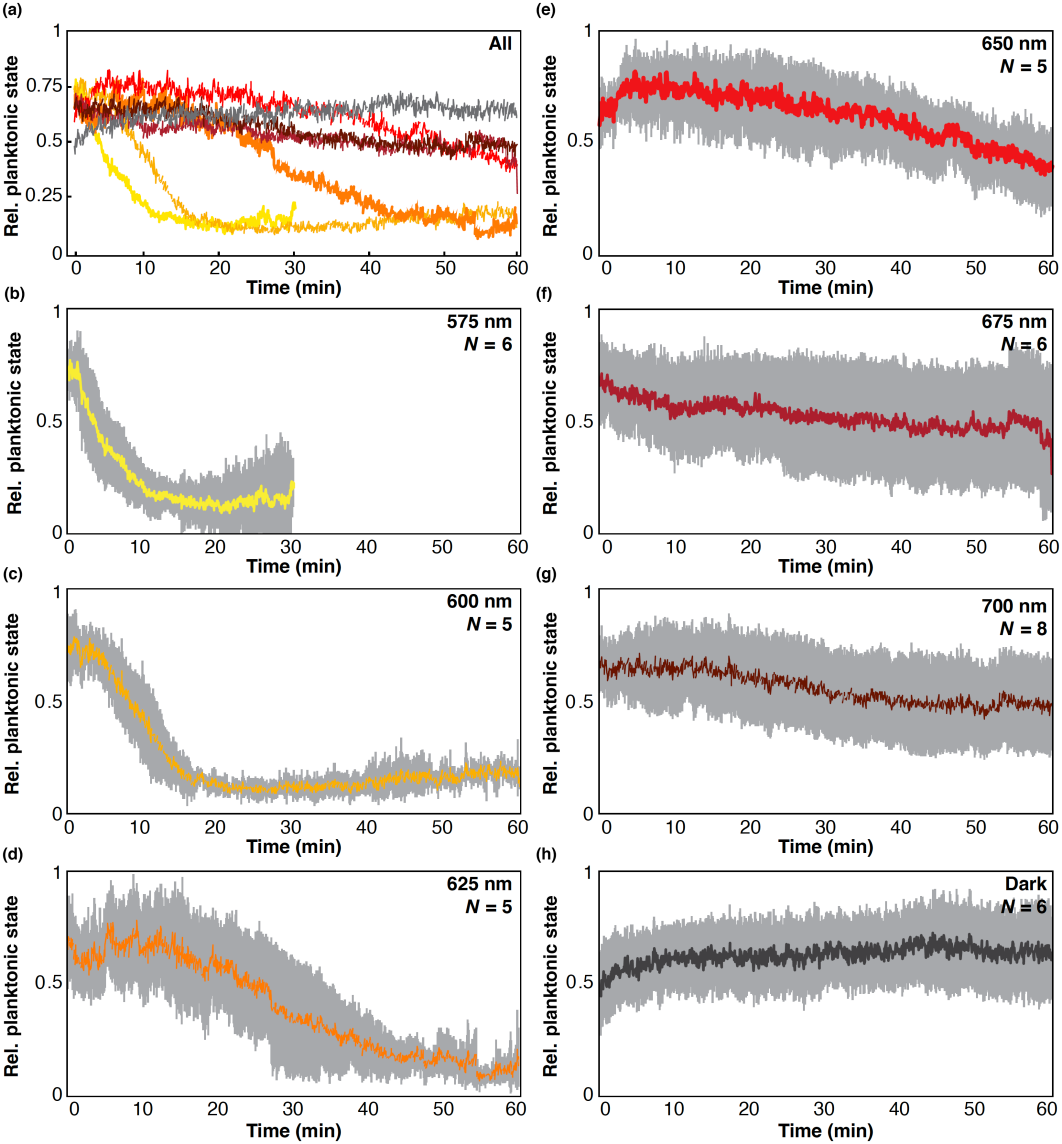
Exiting planktonic state is slower as wavelength increases. (a) All averaged responses over time for long wavelengths overlaid for comparison. Colors of individual lines correspond to colors and wavelengths in (b–h). Averages are taken for at least five experiments per wavelength. (b–h) The same data as in (a), but data for each wavelength are shown individually with 95% confidence intervals. Each wavelength is labeled in each graph and colors correspond to colors in (a).

For longer wavelengths (575–700 nm), changes in behavioral responses and technical limitations required us to modify the experiment (see Section 2). We first noticed response time (time to lowest planktonic state) increased with longer wavelengths making it a challenge to analyze the data in 10‐min trials. We therefore increased trial time for these wavelengths, and to visually compare the change in responses with longer wavelengths we show the average relative planktonic swimming level over time at each wavelength (Figure 7). Downward swimming behavior at 575 nm begins immediately and has the steepest slope of the longer wavelengths, while at 600 nm, animals take longer to respond, and the slope is shallower (Figure 7a–c). At 625 nm, animals take even longer to respond but still settle within 1 h (Figure 7d). At 650, 675, and 700 nm, animals did not reach the lowest planktonic levels seen at wavelengths between 430 and 550 nm, although the slopes were trending downward (Figure 7e–g). In complete darkness, no change was noted after an hour (Figure 7h).

**FIGURE 8:**
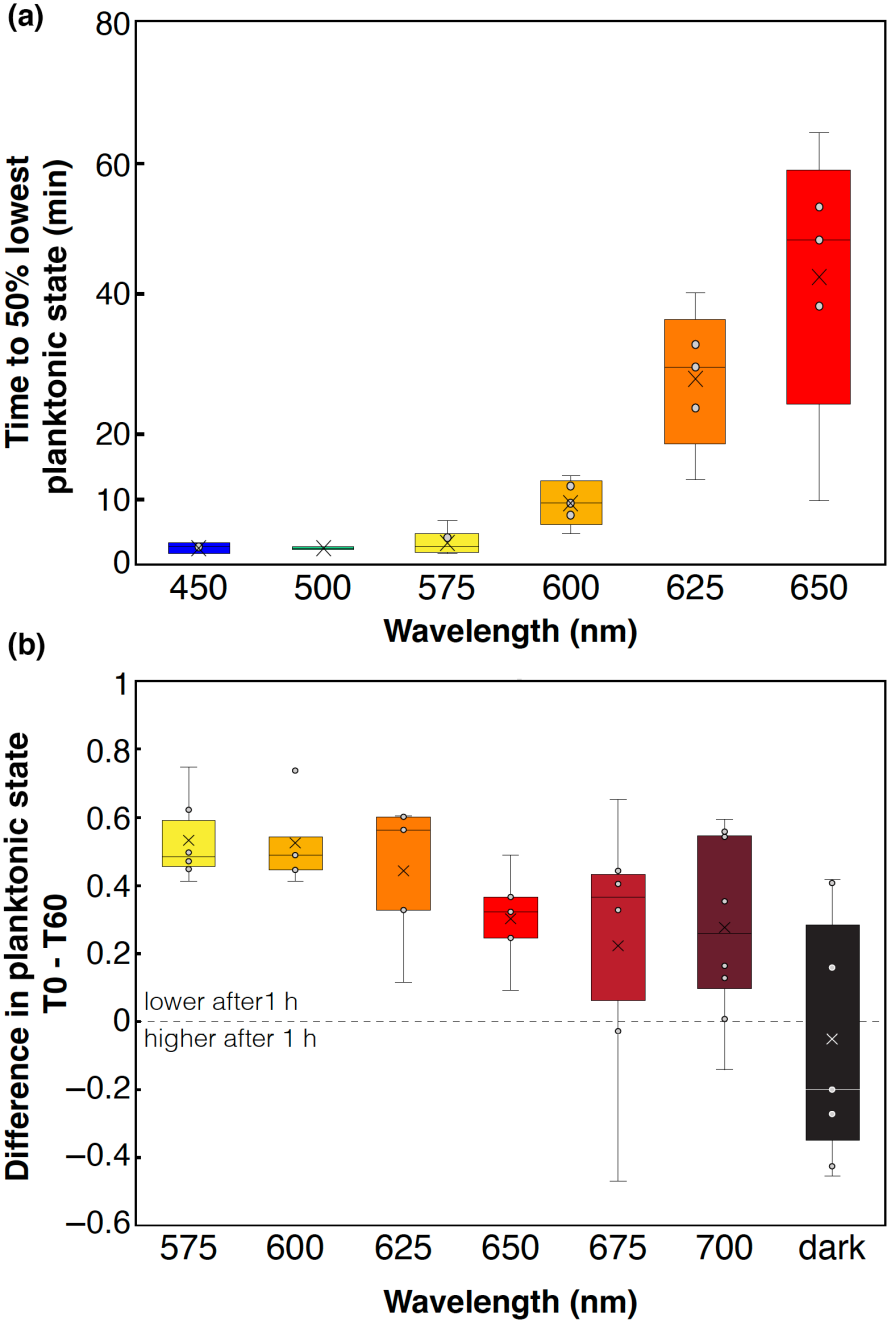
Exiting planktonic phase slows and resembles dark behavior at longer wavelengths. (a) Median time to reach 50% of the lowest planktonic state is plotted from sigmoid curve predictions (see Section 2, Appendix S2). As wavelengths increase after 550 nm, the response time of exiting the planktonic state tends to increase and becomes more variable. For significance values see Table 4. (b) Data longer than 650 nm and dark data could not be fit with sigmoid curves so data were analyzed by measuring the difference in initial swimming activity and activity after 60 min. More positive values signify more larvae exited the planktonic phase, while negative values mean more larvae were in the water column at the end of the trial than at the beginning. A zero value would mean no change in between initial and final levels of planktonic swimming. For significance values see Table 5.

We wanted to quantify the differences in planktonic swimming levels over longer wavelengths to better characterize the behavioral response. To do so, we fit data from each experiment with a sigmoidal function, and identified the inflection point (half‐time to lowest planktonic state) (See Appendix S2 for visualizations of curve fits). We then compared each long wavelength time and two wavelengths from the short wavelength dataset (Figure 8a). We found that timing to lowest planktonic state is not significantly different between 450, 500, and 575 nm (Table 4). There is a significant increase in time from 575 to 625 nm, while 625 and 650 nm are not significantly different (Table 4). Despite no significance, 650 nm appears to continue to increase in response time, but the variance in the data also increases with increasing wavelength (Figure 8a). Without reliable responses, we were unable to fit sigmoid curves to wavelengths longer than 650 or in the dark. To try to quantify differences in responses across all long wavelengths we instead took the difference in planktonic swimming level at the beginning of the hour trial period and the end of the hour for each wavelength (Figure 8b). The trend of the data shows that as wavelengths increase, the difference between swimming levels at start and end time decreases and variability in the data increases, with dark activity being the most variable. In darkness, the median of the data shows a slight *increase* in planktonic swimming after 1 h, though this is highly variable across experiments (Figure 8b). Despite these trends, the only significant difference in these data is between 575 nm and dark trials (Table 5).

## 4 DISCUSSION

Our results indicate that swimming larvae of *N. vectensis* can detect and respond to a broad range of wavelengths, from at least 315 to 625 nm. Larvae do not exhibit phototaxis, as they neither swam toward or away from the light in any wavelength tested. Instead, larvae switch between two behavioral states, either increasing planktonic swimming which may promote dispersal or exiting the planktonic phase, which may be associated with substrate finding for metamorphosis. Peak plantonic swimming levels are seen at wavelengths from 315 to 400 nm, while a transition toward exiting the planktonic state is seen between 415 and 430 nm. From 430 nm up to at least 625 nm animals exit the planktonic phase by seeking the bottom of the cuvette and continue to swim at the bottom once they reach it. At wavelengths from 650 to 700 nm, animals may have some weak response to light stimuli but the quantified behavior is indistinguishable from dark trials, suggesting this is the limit of larval sensitivity. Despite no elaborated visual organ, *N. vectensis* planulae are sensitive to a very broad range of wavelengths. This surprising finding highlights the need for more sophisticated studies of eyeless light‐sensing systems.

The range of wavelengths sensed by *N. vectensis* larvae suggests, assuming opsin‐based light detection, that multiple opsins could be involved in this behavior. A single rhodopsin could peak in the blue region of the spectrum and potentially absorb light at low levels from UV to red wavelengths, but this would be unlikely to mediate these distinct behaviors over the entirety of wavelengths in our dataset. For instance, based on measurements from *Acropora* and rhodopsin template estimates, a blue‐peaking rhodopsin (Acropsin 6) induced no measurable cellular response much past 575 nm (Govardovskii et al., 2000; Mason et al., 2023; Stavenga, 2010). It is possible that the β‐band could allow *N. vectensis* rhodopsins to absorb photons in the UV range, and measured rhodopsin absorbance spectra can have long tails into red wavelengths. However, with a single opsin type, there is no way to distinguish a similarly low response from either the short‐ or long‐wavelength tail of the opsin absorption spectrum; the cellular response would be identical, and it would be impossible to switch behavior based only on this signal.

We have shown that neither behavior is simply induced by a lack of sensitivity to short or long wavelength light (Figure 6). Rather, animals exposed to darkness or wavelengths longer than 625 nm maintain their previous swimming state. Furthermore swimming down is an active behavior driven by longer wavelengths, not a passive side effect due to the lack of a salient signal. Based on the behavioral evidence and opsin absorbance spectra we suggest two and possibly three opsins could be needed to cover this broad sensitivity range. Distinct opsins may be controlling the state of ciliary beating resulting in high planktonic swimming level or substrate‐seeking behavior. Each state can be maintained over a long period of time until a new light stimulus causes a change in cilia beating direction or timing. It is possible animals orient themselves through gravitaxis or perhaps particular patterns of ciliary beating have been selected for that lead to better fitness outcomes under particular light environments, requiring no sensory input for the net outcome. A transition where two opsins may be sending conflicting signals could be between 415 and 430 nm, where some combination of larvae swim up and some swim down. We identified intermediate planktonic swimming levels at these wavelengths, and there could be an individual choice to swim up or down that in aggregate results in intermediate levels in the population as a whole. It is unclear whether this response is specific to each individual and is fixed, or if an individual has some rate of changing its response, leading to this intermediate measured activity. Future experiments could test this with fewer individuals to be able to track them individually. Future directions will address which opsins are involved through targeted CRISPR/Cas9 knockouts and behavioral assays like those established in this study. From previous work, one opsin, *NvASOII‐8b*, is shown to be highly expressed in the sensory apical organ at larval stages (McCulloch et al., 2023). This could be responsible for part of the wavelength‐specific response.

At wavelengths longer than 550 nm, response time increases as wavelength increases. This could be because the putative long‐wavelength absorbing rhodopsin inducing the low planktonic state is less able to absorb photons at these long red and near‐infrared wavelengths. Thus, despite the same total intensity of stimulus, longer wavelengths are in effect dimmer and the behavioral response is therefore less sensitive. Although, not significantly different, the variances of the responses at 650, 675, and 700 nm, do appear different from complete darkness, suggesting there is potentially some sensitivity even to 700 nm light. At all longer wavelengths (625–700 nm), and in the dark, there were at least one or two experiments where animals settled quickly. In the dark, we know animals eventually will settle over a 24‐h period, which could be due to a prolonged lack of any stimulus or so some other non‐light‐based sensory cue that leads to settling over longer time periods in the dark.

It is unknown why the sensitivity and robustness of this behavioral switch across such a broad range of wavelengths in an eyeless larva would be required. Many eyeless marine species have a UV avoidance response, however these are often not sensitive to red light (Brodrick & Jékely, 2023). One explanation is that these are shallow water species and are therefore subject to the full range of wavelengths from the sun (Pope & Fry, 1997; Smith & Baker, 1981). The larval stage is the only dispersal stage in the lifecycle of *N. vectensis* before metamorphosing into a sessile polyp. It could be that short wavelength light in shallow water is itself damaging to the polyp, or is an indicator of a harsh intertidal zone that would dry out polyps that are exposed at low tide. In coastal shallow nutrient‐rich waters, UV light is almost completely attenuated within a meter or two below the surface, and longer red wavelengths are attenuated within 5 or 10 meters, while blue‐green light penetrates much further (Levine & MacNichol, 1982). In the presence of blue, green, or even red wavelengths and the absence of UV/violet, it may be beneficial to swim down and find suitable substrate on which to undergo metamorphosis. Vertical migration in estuaries is a well‐known behavior in plankton that serves for retention in favorable environments or dispersal via advection via tidal currents (Bilton et al., 2002; Kimmerer et al., 2014; Simons et al., 2006). Despite no observed phototaxis, swimming in the water column may allow wave and tidal action to disperse *N. vectensis* larvae, while staying at the substrate in favorable light environments may avoid these external forces, aiding retention of larvae in desirable environments for metamorphosis.

Although the visual systems of eyeless animals have received less attention than their eyed relatives, our work shows that light‐sensing is important and can still be part of a complex trait by these eyeless species. We note that detailed characterization of sensory systems and species previously regarded as “simple”, such as anthozoan cnidarians, can reveal overlooked complexity in sensory traits and behavior. Further work will investigate the molecular‐genetic basis for this and other behavioral traits to get a fuller comparative picture of visual system evolution. Although more work needs to be done, elucidating part of the *N. vectensis* visual system is an important step in broadening our understanding of the evolution of complex traits like animal eyes.

## AUTHOR CONTRIBUTIONS

**Emma Lilly:** Data curation (lead); formal analysis (equal); funding acquisition (equal); investigation (lead); software (equal); writing – review and editing (lead). **Meghan Muscala:** Data curation (equal); formal analysis (lead); funding acquisition (equal); investigation (equal); software (equal); writing – original draft (lead). **Camilla R. Sharkey:** Formal analysis (equal); methodology (equal); writing – review and editing (equal). **Kyle J. McCulloch:** Conceptualization (lead); data curation (equal); formal analysis (equal); funding acquisition (equal); investigation (supporting); methodology (lead); project administration (lead); resources (lead); software (lead); supervision (lead); validation (lead); visualization (lead); writing – review and editing (equal).

## CONFLICT OF INTEREST STATEMENT

The authors declare no conflicts of interest.

## Supporting information


Appendices S1–S2

## Data Availability

Trackmate raw data for all swimming activity used in this study is available in Dryad at https://doi.org/10.5061/dryad.wdbrv15vs. Videos are available upon request. All other data in this study are included in the figures or as appendices.
