# Supplementary material for: Larval swimming in the sea anemone Nematostella vectensis is sensitive to a broad light spectrum and exhibits a wavelength‐dependent behavioral switch
Source: Ecol Evol. 2024 Apr 15;14(4):e11222. doi: 10.1002/ece3.11222 (PMC11019245; doi:10.1002/ece3.11222)

Appendix 1: Number of replicates for short and long wavlength experiments

Wavelength	N experiments
315	5
320	5
325	6
330	7
335	4
340	3
345	5
350	5
355	7
360	6
365	7
370	5
375	7
380	6
385	5
390	4
395	4
400	12
405	9
410	6
415	5
420	11
425	7
430	3
435	4
440	4
445	4
450	5
455	8
460	6
465	7
470	4
475	5
480	4
485	6
490	6
495	6
500	5
505	7
510	5
515	7

	520	9
	525	4
	530	4
	535	7
	540	3
	545	8
	550	10
	575	6
	600	5
	625	5
	650	5
	675	6
	700	8
dark		6

230706 - 575nm

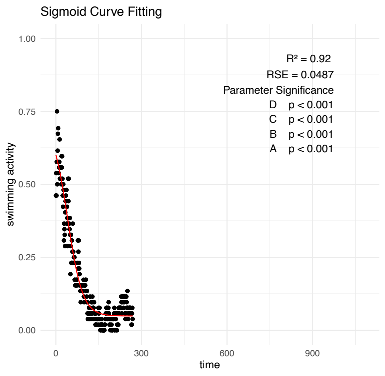

230523 - 575nm

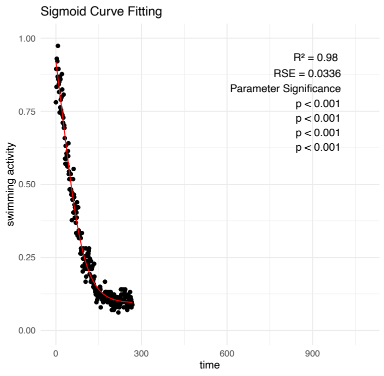

230515 - 575nm

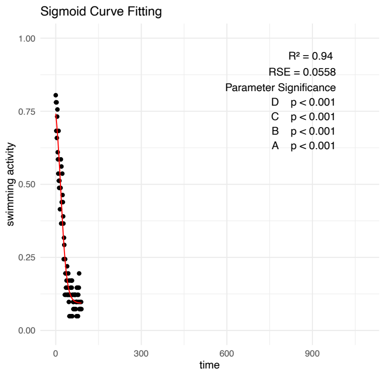

230807 - 600 nm

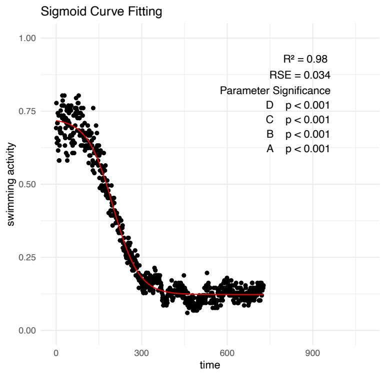

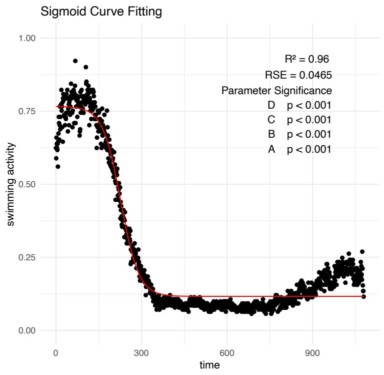

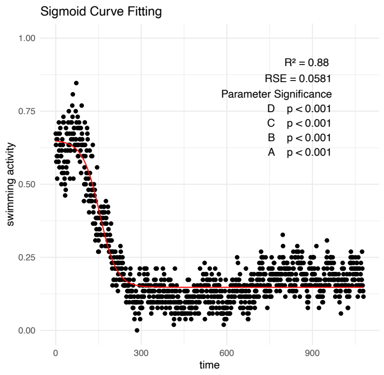

230710 - 625 nm

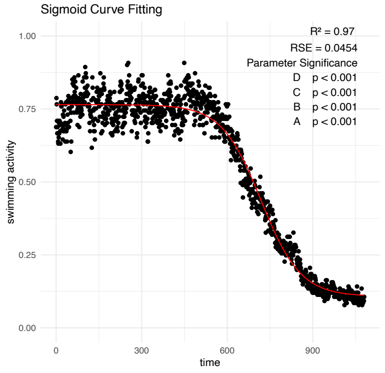

230913 - 625 nm

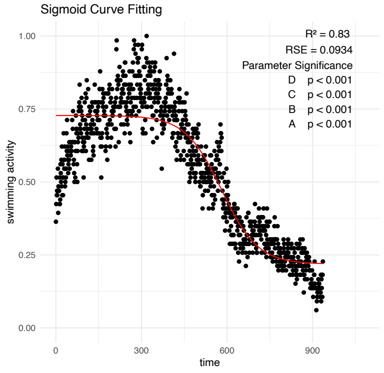

230807 - 625 nm

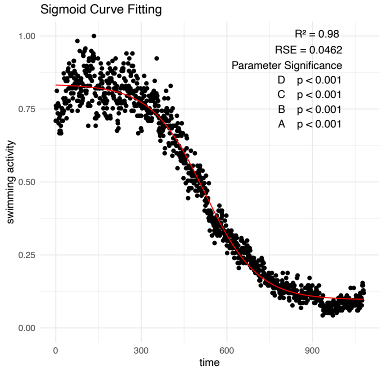

230817 - 650 nm **no fit**

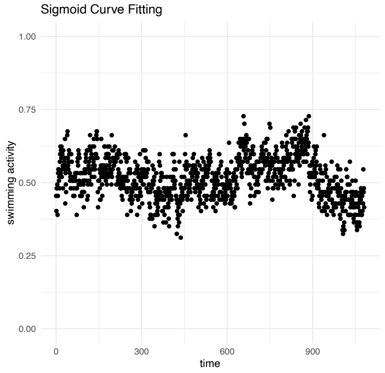

230807 - 650 nm

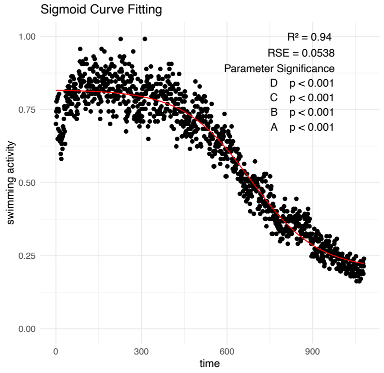

230629 - 650 nm

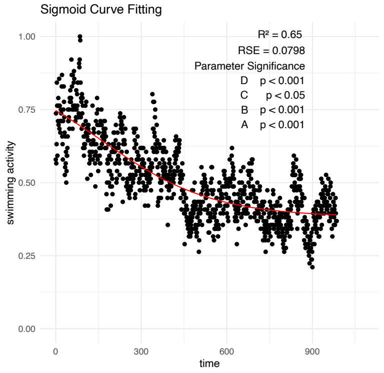

Exp1 675 nm

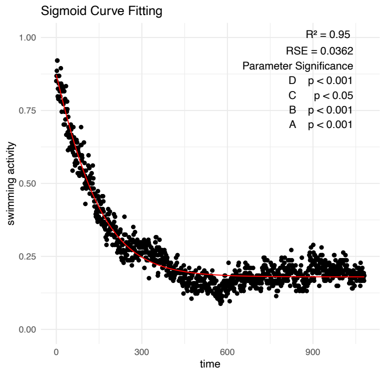

Exp6 675 nm, **no fit**

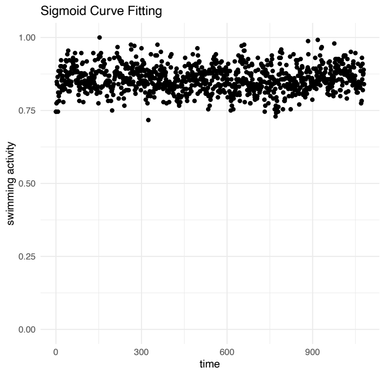

Exp5 675 nm

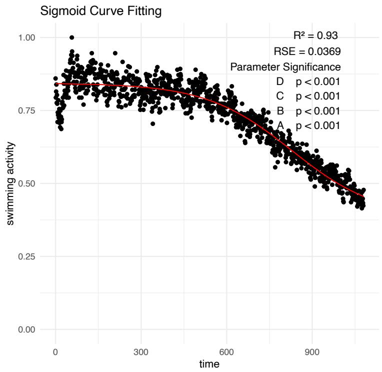

Exp5 - 700 nm **no fit**

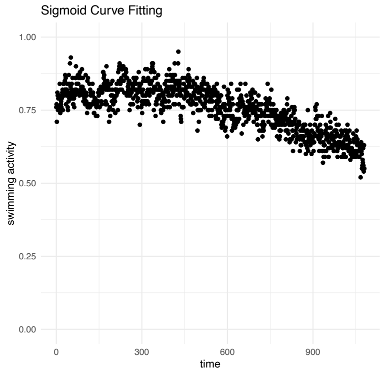

Exp6 - 700 nm

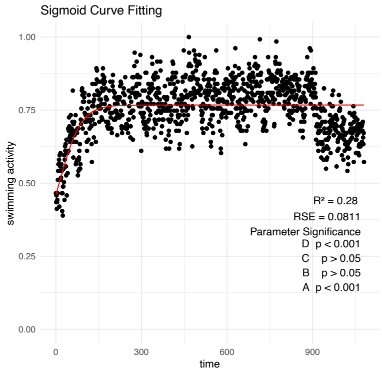

230817 - 700 nm

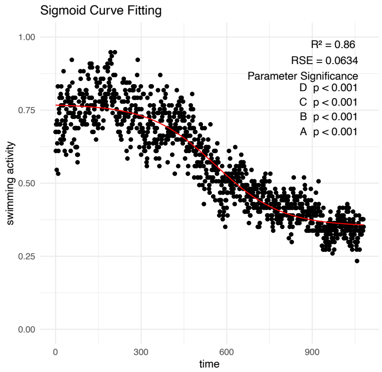

Exp3 - dark

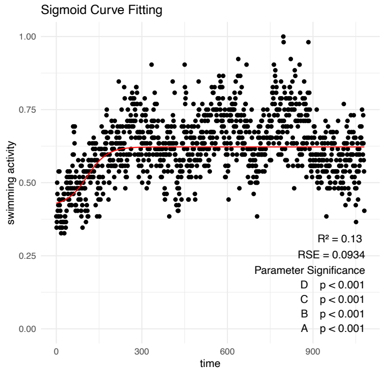

Exp5 - dark

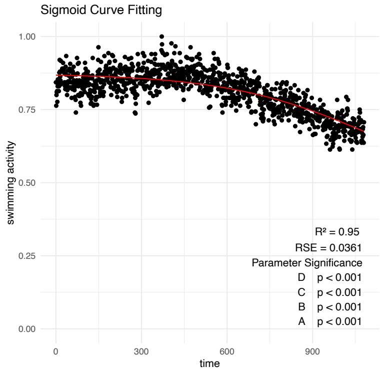

Exp7 - dark

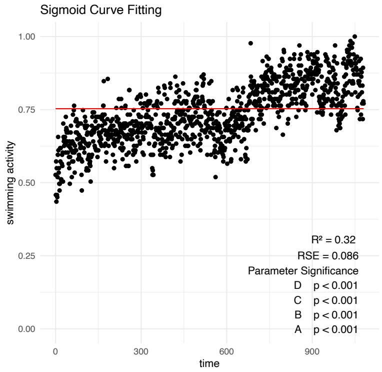

Supplement: Supplementary file 1 — Appendices S1–S2 [file ECE3-14-e11222-s001.pdf]
